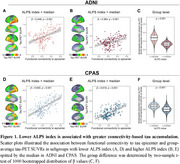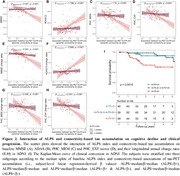# Impaired glymphatic function exacerbates tau propagation and the effect of tau pathology on cognitive decline in Alzheimer's disease

**DOI:** 10.1002/alz70856_102663

**Published:** 2025-12-25

**Authors:** Ying Luan, Ying Cui, Jie Wang, Qi Huang, Xiaoxie Mao, Binyin Li, Fang Xie, Alzheimer's Disease Neuroimaging Initiative

**Affiliations:** ^1^ Zhongda Hospital, School of Medicine, Southeast University, Nanjing, China; ^2^ Huashan Hospital, Fudan University, Shanghai, Shanghai, China; ^3^ Department of Nuclear Medicine & PET Center, Huashan Hospital, Fudan University, Shanghai, Shanghai, China; ^4^ School of Medicine, Xiamen University, Xiamen, Fujian, China; ^5^ Ruijin Hospital affiliated to Shanghai Jiaotong University School of Medicine, Shanghai, Shanghai, China

## Abstract

**Background:**

Tau protein aggregation is hypothesized to spread across interconnected neurons, where pathological tau is released from the presynaptic terminal via exocytosis and subsequently taken up by the postsynaptic neuron via endocytosis. Emerging evidence suggests that the glymphatic system, a perivascular network, plays a crucial role in waste clearance within the interstitial fluid. Impaired glymphatic clearance has been associated with tau aggregation, potentially accelerating disease progression. Whether glymphatic function modulates inter‐neuronal tau propagation remains unclear.

**Method:**

We included 173 participants and 303 participants from Alzheimer's Disease Neuroimaging Initiative (ADNI) and Chinese Preclinical Alzheimer's Disease Study (CPAS) respectively, with the availability of baseline T1‐weighted MRI, ^18^F‐florbetapir or ^18^F‐florbetaben amyloid‐PET, diffusion tensor imaging (DTI), 18F‐Flortaucipir tau‐PET and cognitive assessments. For ADNI participants, longitudinal clinical assessments were also collected. Diffusion tensor image analysis along the perivascular space (DTI ALPS) index was calculated to assess the movement of water molecules along the perivascular spaces. The participants were separated into two subgroups by the median split of DTI ALPS. Tau epicenters were defined as regions with the top 10% tau‐PET SUVRs, and the association between normative functional connectivity to tau epicenter and tau‐PET levels in connected regions was assessed. Mixed‐effect models assessed interactions between ALPS index and epicenter connectivity on tau accumulation. The effects of ALPS and connectivity‐based tau accumulation (subject‐level regression‐derived β values) on cognition and clinical progression was further evaluated.

**Result:**

We found higher connectivity‐based tau accumulation in participants with lower ALPS indices (Figure 1). Subjects with lower ALPS showed greater tau‐PET SUVRs along epicenter connectivity pattern. As shown in Figure 2, lower ALPS is associated with poor baseline cognitive performance and faster cognitive decline rates as a function of connectivity‐mediated tau accumulation. Kaplan‐Meier analyses showed faster clinical progression in subgroups with lower ALPS and higher tau accumulation.

**Conclusion:**

Our findings suggested that impaired glymphatic system might plays an adverse role on cognitive function and clinical progression via alleviating tau clearance from the inter‐neuronal space, which may also facilitate the tau spread. The current results provide novel understanding of clearance mechanism of tau pathology and encourage future work to develop glymphatic system‐targeted therapy for AD.